# Urinary Biomarkers for Early Detection of Kidney Injury Following Extracorporeal Shock Wave Lithotripsy: A Systematic Review

**DOI:** 10.7759/cureus.90688

**Published:** 2025-08-21

**Authors:** Abhinav Singhal, Maanya Bhardwaj, Gaurika Bhardwaj, Nada Humayun-Zakaria

**Affiliations:** 1 Urology, University Hospitals Birmingham NHS Foundation Trust, Birmingham, GBR; 2 Major Trauma, Nottingham University Hospital NHS Trust, Nottingham, GBR; 3 Critical Care, Chelsea and Westminster Hospital NHS Foundation Trust, London, GBR; 4 Urology, University Hospitals Birmingham NHS Foundation Trust, Burmingham, GBR

**Keywords:** eswl, extracorporeal shock wave lithotripsy, kidney injury molecule-1 (kim-1), kidney stones, nephrolithiasis, neutrophil gelatinase-associated lipocalin (ngal), shock wave lithotripsy, ureteric stones, urinary biomarker, urinary calculi

## Abstract

Extracorporeal shock wave lithotripsy (ESWL) is a commonly used, non-invasive intervention used for the treatment of urinary stones, however, it carries the risk of causing renal injury. Renal function parameters such as serum creatinine and estimated glomerular filtration rate (eGFR) often fail to detect early or subclinical damage. This systematic review evaluates the diagnostic utility of urinary and plasma biomarkers, particularly neutrophil gelatinase-associated lipocalin (NGAL), cystatin C, KIM-1, and IL-18, for early detection of renal injury following ESWL.

Following Preferred Reporting Items for Systematic Reviews and Meta-Analyses (PRISMA) guidelines, databases such as PubMed, EMBASE, CENTRAL, and SCOPUS were used to identify studies published between 1st January 2015 to 15th June 2025. Eligible studies included adults undergoing ESWL which reported pre- and post-procedure urinary biomarker levels. Eight studies met the inclusion criteria, including two randomized controlled trials, four prospective cohort studies, and two controlled observational studies. Risk of bias was assessed using RoB 2.0, ROBINS-I, and the Newcastle-Ottawa Scale.

Across 412 participants, NGAL was the most frequently evaluated biomarker, showing early and significant increases within six to 12 hours post-ESWL, often before changes in serum creatinine or eGFR. Cystatin C also rose consistently post-procedure and proved more sensitive than creatinine in some studies. KIM-1 and IL-18 showed high diagnostic accuracy (area under the curve (AUC) up to 0.951) for tubular injury. However, heterogeneity in ESWL protocols, sample timing, and study populations limited comparability. Some studies suggested that NGAL responses may be affected by factors like hydration or stone location.

Urinary biomarkers including NGAL, cystatin C, and KIM-1 offer promising, non-invasive tools for early detection of renal injury after ESWL. Traditional markers such as serum creatinine often remain unchanged immediately post-ESWL, and novel biomarkers can reflect subclinical tubular and glomerular damage within hours of treatment. Despite these encouraging findings, variability across studies and lack of long-term outcome data warrant further standardized research. Future studies should assess biomarker kinetics in relation to ESWL parameters and their prognostic value for sustained renal dysfunction.

## Introduction and background

Urinary stones are a common condition with an annual incidence of one to two cases per 1000 people [1]. Recurrence rates are particularly high, reaching up to 50% within 10 years [1]. Several factors influence the management of urinary stones, such as size, severity of symptoms, location of the stone, and age of patients. Extracorporeal shock wave lithotripsy (ESWL) is a widely used, non-invasive surgical technique to break stones in the kidney and ureter. The mechanism involves generation of shockwaves by an external source which are transmitted with the help of a coupling medium [1]. These shockwaves converge to the point of maximum intensity, by passing through the soft tissues and focusing on the stone to cause fragmentation [1]. ESWL is indicated for patients with kidney stones measuring 4-20 mm, particularly those located in the renal pelvis, upper or middle calyces, or proximal ureter [2]. Suitable candidates should have a functioning kidney, no distal obstruction, and no active urinary tract infection. ESWL can cause varying degrees of renal injury such as haemorrhage, septic shock, ureteral obstruction, development or exacerbation of hypertension, perinephric hematoma or tubular damage [3]. Early detection of these injuries is crucial as it allows timely interventions to minimize permanent loss of renal function. It also guides post-procedure care and influences decisions regarding future management such as repeating ESWL or choosing alternative treatment methods in patients with compromised renal function. Although serum creatinine and estimated glomerular filtration rate (eGFR) are routinely used to assess renal function, they are often insensitive, nonspecific, and may only rise after significant kidney damage has occurred [3].

Novel biomarkers have been investigated for their ability to detect renal dysfunction earlier and more accurately. Biomarkers such as cystatin C and neutrophil gelatinase-associated lipocalin (NGAL) have demonstrated clinical utility in detecting renal dysfunction earlier than traditional serum creatinine or urea-based assessments [4]. Cystatin C is a low-molecular-weight protein freely filtered by the glomeruli and reabsorbed in the proximal tubules, making it a more reliable marker of eGFR than creatinine, particularly in individuals with altered muscle mass or those at early stages of chronic kidney disease (CKD) [4,5]. Its levels are less influenced by age, sex, and dietary intake, offering improved sensitivity in detecting mild renal impairment. NGAL, by contrast, functions as an early marker of tubular injury and can detect acute kidney injury (AKI) within two to four hours of insult, significantly earlier than serum creatinine, which typically rises after 24-48 hours [5,6]. While both biomarkers require specific immunoassay-based testing, which may not be readily available in all routine laboratories, they provide a valuable opportunity for early intervention and risk stratification in patients at high risk of renal injury [6]. Their inclusion in clinical decision-making algorithms could enhance diagnostic precision and reduce the incidence of delayed AKI diagnosis. This systematic review aims to evaluate the use of urinary biomarkers such as cystatin C and NGAL for the early detection of renal injury following ESWL.

## Review

Methods

**Figure 1:**
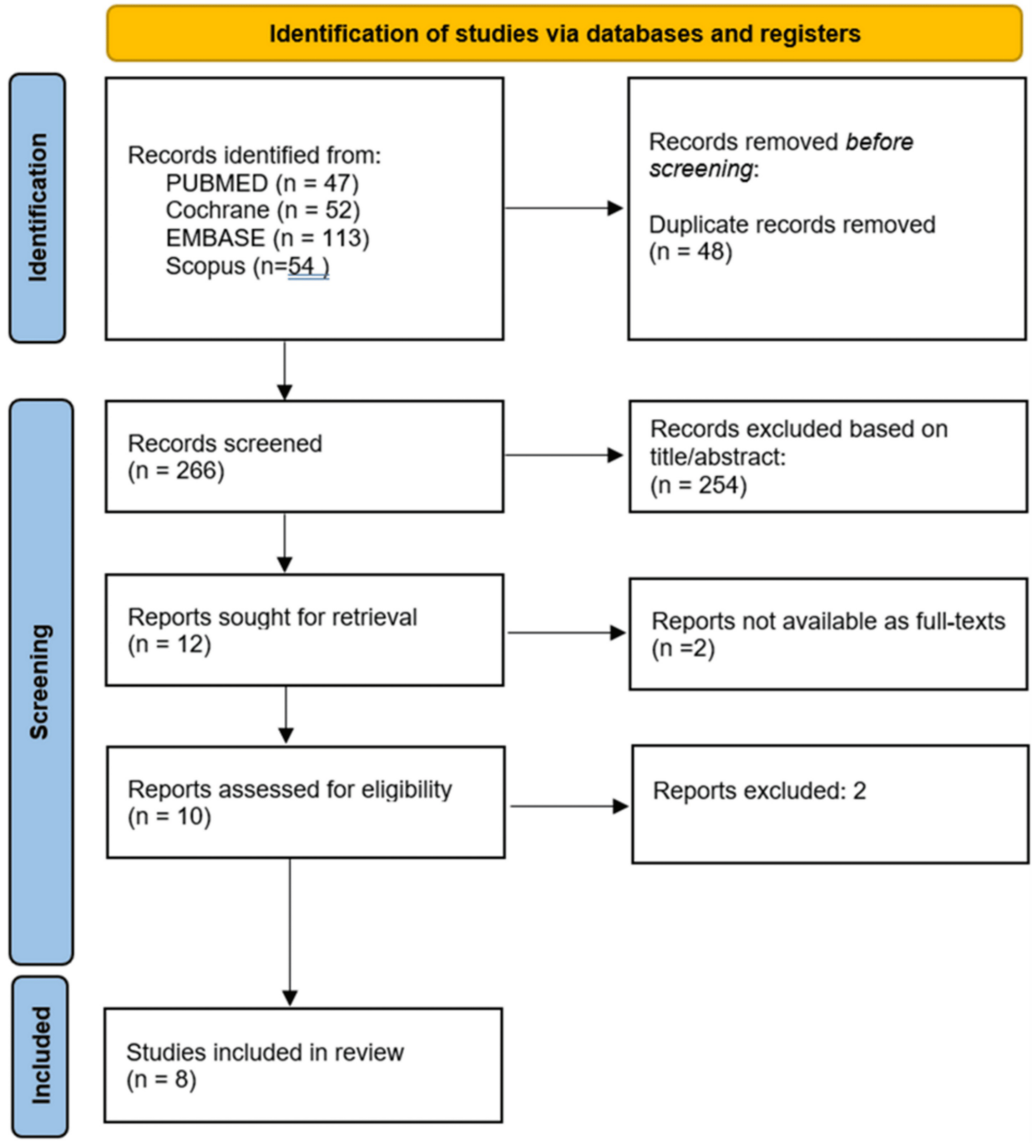
Preferred Reporting Items for Systematic Reviews and Meta-Analyses (PRISMA) flow diagram for identifying studies for inclusion in systematic review

This systematic review was conducted in accordance with the Preferred Reporting Items for Systematic Reviews and Meta-Analyses (PRISMA) guidelines (Figure 1) [7]. A comprehensive search was conducted in the following electronic databases PubMed/MEDLINE, EMBASE, Cochrane Central Register of Controlled Trials (CENTRAL) and SCOPUS. The search included all articles published from 2015 to 2025. A structured search strategy was developed using keywords and Medical Subject Headings (MeSH) terms. The search terms included combinations of extracorporeal shock wave lithotripsy, shock wave lithotripsy, ESWL, urinary biomarker, neutrophil gelatinase-associated lipocalin, KIM-1, cystatin C, acute kidney injury, renal injury or renal function. Full search strategies used for the databases are available in Appendix A. Studies were selected according to the inclusion and exclusion criteria outlined in Table 1. Titles and abstracts were screened independently by three reviewers using the inclusion and exclusion criteria. Full texts were retrieved for eligible studies and assessed for final inclusion [7].

**Table 1:** Exclusion and inclusion criteria for the studies ESWL: extracorporeal shock wave lithotripsy

	Inclusion	Exclusion
Population	Paper only includes adults >18 years Patients undergoing ESWL for nephrolithiasis or ureterolithiasis	Any other surgery Paediatric patients (<18 years)
Study design	Randomised controlled trials Prospective or retrospective cohort studies Case control studies Case series >20 patients	Case reports Case series < 20 patients Abstracts
Language	Papers in English	Non-English language
Time frame	Papers from 2015-2025	Papers outside this timeframe
Outcomes	Urinary biomarker levels before and after ESWL	No relevant clinical data

Data was extracted independently by three reviewers using a standardized form. The following data was collected: study characteristics, population details, intervention details, comparator details, outcome measures, key findings and conclusions. The methodological quality of included studies was assessed using the Cochrane Risk of Bias 2.0 tool [8] for randomized controlled trials, ROBINS-I for non-randomized studies [9] and the Newcastle-Ottawa scale (NOS) for cohort and case control studies [10]. Each domain was rated as low, moderate, or high risk of bias. Disagreements between reviewers were resolved through discussion and re-examination of the eligibility criteria. Persistent discrepancies were adjudicated by a fourth reviewer to ensure consensus. Due to expected heterogeneity in study designs and outcome measures, a narrative synthesis was conducted. Studies were grouped based on intervention characteristics and outcome type.

Results

A total of 226 records were identified through database searches, and 33 full-text articles were reviewed in detail. Based on the inclusion and exclusion criteria, eight studies published between 2015 and 2025 were included in the final synthesis. These comprised two randomized controlled trials (RCTs), four prospective observational studies, and two controlled cohort studies. The included studies collectively enrolled 700 participants, with individual study sample sizes ranging from 15 to 320. All studies evaluated renal injury following a single or multiple sessions of ESWL, using urinary or plasma biomarkers for early detection of AKI. Commonly evaluated biomarkers included NGAL, cystatin C, KIM-1, and IL-18. A detailed summary of included studies is provided in Table 2.

**Table 2:** Characteristics of included studies ESWL: extracorporeal shock wave lithotripsy, eGFR: estimated glomerular filtration rate, NGAL: neutrophil gelatinase-associated lipocalin, uNGAL: urinary NGAL, pNGAL: plasma NGAL, NAG: N-acetyl-beta-D-glucosaminidase, RCT: randomized controlled trial, AUC: area under the curve

	Study Design	Sample Size	Biomarker(s) Evaluated	Key Findings	Diagnostic Performance	Risk of Bias
Milišić et al [11]	Prospective controlled study	62	Urinary NGAL	↑ uNGAL by 126% at 6h and 584% at 12h; negative correlation with eGFR	AUC = 0.694; Sensitivity 60.6%, Specificity 75.0%	Low
Dzięgała et al. [12]	Prospective controlled study	62	Urinary NGAL	↑ uNGAL at 6h and 12h; eGFR dropped 15.3% at 12h; partial recovery by 3 months	Not reported; Sensitivity 60.6%, Specificity 75.0%	Low
Hughes et al [13]	Prospective pilot study	15	Plasma & Urinary NGAL	↑ pNGAL and uNGAL post-ESWL; no change in creatinine; early renal injury marker	Not reported	Moderate
Turan et al. [14]	RCT	94	Cystatin C	↑ Cystatin C and ↓ eGFR after ESWL; more sensitive than creatinine	Not reported	Low
Tawfick et al [15]	Prospective observational	60 ESWL, 20 controls	KIM-1, NGAL, IL-18, Cystatin C (mRNA)	↑ expression of all markers post-ESWL; KIM-1 and NGAL were most predictive	KIM-1: AUC 0.951; NGAL: AUC 0.903	Low–Moderate
Vittori et al [16]	Prospective observational	24	NGAL, NGAL/Creatinine ratio	↑ uNGAL at 3h post-ESWL	Not reported	Moderate–High
Iskhakova & Gilmanov [17]	Prospective cohort	35 ESWL, 14 controls	NGAL, β2-Microglobulin, Cystatin C, Microalbumin	↑ NGAL after each session; AUC 0.77–0.80; NGAL outperformed other biomarkers	NGAL: AUC 0.77–0.80	Moderate
Ng et al. [18]	Randomized controlled trial	320	Urinary NGAL, MA, IL-18 and NAG	↑ in MA, IL-18 and NAG, however, no increase in NGAL	Not reported	Low

Seven of the eight studies reported data on urinary or plasma NGAL. Both urinary and plasma NGAL levels consistently increased within hours following ESWL, often preceding any detectable change in serum creatinine or eGFR. Milišić et al. found a 584% increase in urinary NGAL at 12 hours post-ESWL (p < 0.001), which negatively correlated with eGFR across follow-up points [11]. Dzięgała et al. [12] and Hughes et al. [13] reported similar trends, confirming urinary NGAL as a sensitive early marker of renal stress. Two studies [14,15] demonstrated that cystatin C rises significantly after ESWL and may offer higher sensitivity than serum creatinine. Turan et al. [14] found cystatin C detected renal function decline at both one and 30 days post-treatment despite stable creatinine levels. Vittori et al. [16] noted that NGAL levels normalized within 30 days, suggesting short-lived injury in most patients. In contrast, Dzięgała et al. [12] found that eGFR remained 10.1% lower at three months, indicating possible prolonged functional impairment.

Tawfick et al. [15] investigated the diagnostic potential of urinary biomarkers following ESWL using a panel of long non-coding RNAs (SBF2-AS1, FENDRR19) and mRNAs (GBP1, NLRP3), and found elevated expression of KIM-1 and IL-18. SBF2-AS1 demonstrated highest sensitivity at 91.7%, followed by FENDRR19 (76.7%), and both GBP1 and NLRP3 (78.3%). These findings suggest that urinary expression of these RNAs rises early after lithotripsy and may serve as sensitive indicators of subclinical AKI. This is particularly relevant given the limitations of conventional markers such as serum creatinine, which often rises only after significant nephron loss [5].

Risk of bias assessment

The risk of bias was assessed using Cochrane’s RoB 2.0 for randomized controlled trials [8], ROBINS-I for non-randomized studies [9], and the Newcastle-Ottawa scale for observational studies [10]. A summary of the assessments is provided in Table 3.

**Table 3:** Summary of the risk of bias assessment NOS: Newcastle-Ottawa scale, ROBINS-I: Risk of Bias in Non-randomised Studies - of Interventions, RoB 2.0: Risk of Bias 2, RCT: randomized controlled trial

Study	Design	Tool Used	Risk of Bias Summary
Milišić et al. [11]	Observational	ROBINS-I	Low – Good control of confounding factors and outcomes
Dzięgała et al. [12]	Controlled	ROBINS-I	Low – Strict exclusion criteria, robust measurements
Hughes et al. [13]	Pilot Study	ROBINS-I	Moderate – small sample size, no stratification by stone size or number of shocks
Turan et al. [14]	RCT	RoB 2.0	Low – Randomized, blinded lab analysis, no missing data
Tawfick et al. [15]	Observational	ROBINS-I	Low to Moderate – RNA biomarkers, no longitudinal follow-up
Vittori et al. [16]	Observational	NOS	Moderate to High – Small sample, no control group
Iskhakova & Gilmanov [17]	Cohort	NOS	Moderate – Small sample, no adjustment for confounders
Ng et al. [18]	RCT	RoB 2.0	Low – Good randomization and biomarker evaluation. Well-designed, with transparent methodology. Single-center study; results may not be generalizable. NGAL unexpectedly unresponsive, not explained by authors

Overall, the quality of evidence is moderate to high. The most consistent limitations were small sample sizes and short follow-up durations, although methodological rigor specifically in biomarker measurement and confounder control was generally strong.

Discussion

This systematic review evaluated the role of urinary biomarkers in detecting early renal injury following ESWL. Across eight studies, multiple biomarkers such as NGAL, cystatin C, KIM-1, IL-18, and N-acetyl-beta-D-glucosaminidase (NAG) were utilized to identify kidney damage. While serum creatinine and eGFR often remained unchanged immediately post-ESWL, several biomarkers showed early and transient elevations, indicating that biomarkers may serve as more sensitive indicators of AKI than traditional renal function parameters.

NGAL as a Central Biomarker

NGAL emerged as the most investigated biomarker in the context of renal injury following ESWL, highlighting its growing recognition as a sensitive indicator of early tubular stress. Its consistent elevation in multiple studies within six to 12 hours post-treatment suggests that NGAL may offer a valuable early signal of subclinical renal damage well before changes in conventional renal markers become apparent. Notably, multiple studies (n=6) reported an inverse correlation between NGAL levels and eGFR, underscoring its potential to predict longer-term renal function decline. For instance, Milišić et al. [11] observed a marked 584% rise in urinary NGAL (uNGAL) at 12 hours post-ESWL, which was significantly associated with a sustained reduction in eGFR at three months. Likewise, Iskhakova and Gilmanov [17] demonstrated high diagnostic accuracy of NGAL, with AUC values exceeding 0.77 following each ESWL session. These findings collectively support NGAL as a promising biomarker for early detection of renal stress and potential injury in patients undergoing lithotripsy, offering opportunities for closer monitoring and timely intervention.

Despite the promising findings, discrepancies in the performance of NGAL were noted. Vittori et al. [16] reported statistically significant increases in NGAL after ESWL, however, eight patients with haematuria (a known marker of renal trauma) showed no increase in NGAL, suggesting limitations with biomarker sensitivity. This highlights the importance of contextualising biomarker data in relation to imaging and clinical symptoms for interpretative accuracy. Additionally, some studies did not identify a direct correlation of NGAL with AKI. Ng et al. [18] found no increase in NGAL post-treatment. In contrast, other biomarkers such as NAG, microalbumin (MA), and IL-18 levels were elevated immediately post-ESWL in all groups, indicating acute renal injury. Given the heterogeneity in biomarker response of NGAL, it may not fully capture the spectrum of renal injury induced by ESWL. A multimodal approach with combined analysis of multiple biomarkers alongside clinical and radiological findings would enhance early detection and characterization of renal injury.

Other Biomarkers: Cystatin C, KIM-1, and IL-18

Cystatin C has shown promise as a mid-term marker of renal dysfunction as evidenced by Turan et al. [14] in a well-controlled RCT, demonstrating its superiority over creatinine in detecting renal dysfunction at one and 30 days post-ESWL. Standardization of equipment, single operator and structured sampling (baseline, day one and day 30) adds strength to their findings. The controlled design enhances reliability of cystatin C and offers compelling evidence to incorporate this biomarker in routine post-ESWL monitoring of AKI.

RNA expression of KIM-1, NGAL, IL-8 and cystatin C illustrated strong diagnostic performance of AKI, particularly of KIM-1 (AUC = 0.951) based on focused assessment of short-term biomarker kinetics [15]. Elevated levels of IL-18 support its role in early injury detection [15,18] immediately post-ESWL following normalization to baseline by day two, showcasing a possible renoprotective effect of treatment pacing.

Although no single biomarker has emerged as definitive, the combined use of NGAL, cystatin C, and KIM-1 offers promise for early, non-invasive detection of ESWL-induced renal injury. Table 4 illustrates a structured comparison of urinary biomarkers highlighting differences in their kinetics, sensitivity, and clinical utility following ESWL. Biomarker trends could inform post-treatment monitoring strategies or even pre-treatment risk stratification. However, given the variability in biomarker responses and the absence of long-term clinical outcome data in most studies, biomarkers should currently be used as adjunctive tools rather than replacements for imaging and clinical judgment.

**Table 4:** Summary of key characteristics, detection kinetics, and clinical considerations for urinary biomarkers investigated following ESWL ESWL: extracorporeal shock wave lithotripsy, GFR: glomerular filtration rate, NGAL: neutrophil gelatinase-associated lipocalin, NAG: N-acetyl-beta-D-glucosaminidase, AUC: area under the curve, AKI: acute kidney injury

Biomarker	Source	Peak Detection Post-ESWL	Sensitivity & Specificity	Limitations	Clinical Applicability
NGAL	Tubular stress protein	6–12 hours	Early AKI detection; AUC >0.77 in multiple studies	Rapid rise; influenced by hematuria or patient variability; best interpreted with clinical context	Useful for early detection of subclinical renal injury; may guide short-term monitoring post-ESWL
Cystatin C	Low-molecular-weight protease inhibitor	1–30 days	Reliable marker of GFR decline	Less useful for very acute injury; minimally affected by muscle mass	Suitable for mid-term monitoring and assessing renal function recovery; complements creatinine
KIM-1	Tubular injury protein / RNA	Immediate	High diagnostic performance (AUC ~0.95)	Mostly RNA/protein research assays; clinical availability limited	Promising research biomarker; not yet widely available for routine clinical use
IL-18	Proinflammatory cytokine	Immediate post-ESWL	Detects early tubular injury	Normalizes quickly; timing critical; may reflect inflammation rather than pure tubular injury	Potential early injury marker; clinical use limited due to rapid normalization and assay availability
NAG	Tubular enzyme	Immediate	Detects acute tubular injury	Non-specific; affected by proteinuria	Can support early injury detection as part of a multimarker panel; not standalone
Microalbumin (MA)	Glomerular/tubular leakage marker	Immediate	Sensitive for acute injury	Non-specific; influenced by baseline proteinuria	Useful for detecting glomerular/tubular leakage; best combined with other biomarkers

Strengths and Limitations of Included Studies

Most studies employed prospective designs and utilised validated biomarker assays with blinded outcome assessment as reported in key trials such as Turan et al. and Ng et al. [14,18]. Both described the randomization procedures in detail, reducing risk of selection bias and enhancing internal validity.

However, heterogeneity across studies limits comparability due to variability in sample size, ESWL energy protocols, and sampling schedules. For instance, Tawfick et al. [15] and Vittori et al. [16] used differing post-procedure sampling windows (2h vs. 12-24h), limiting comparability.

Potential confounding variables like patient hydration status (noted in Vittori et al. [16]) and comorbidities were inconsistently addressed. Additionally, few studies stratified results by stone location or baseline renal function, both of which can influence outcomes. Standardization in these areas is needed to strengthen future studies to allow more robust comparisons.

Future Recommendations

Future research should focus on standardizing biomarker measurement, evaluating their predictive value for long-term renal function, and assessing their role in guiding personalized treatment protocols. In addition, studies are required to determine changes in NGAL values in relation to the different frequencies of the shock waves.

## Conclusions

This systematic review highlights the potential utility of urinary and plasma biomarkers, particularly NGAL, cystatin C, KIM-1, IL-18, NAG, and microalbumin, in detecting early renal injury following ESWL. While conventional markers such as serum creatinine often remain unchanged immediately post ESWL, these biomarkers can reflect subclinical tubular and glomerular damage within hours of treatment. Among them, NGAL and cystatin C showed the most consistent early elevation and diagnostic value, although responses varied depending on patient factors, and treatment protocols. Use of urinary biomarkers after ESWL may enable early identification of subclinical tubular stress, allowing timely interventions such as closer monitoring, hydration, or adjustment of subsequent procedures. Early detection could reduce the risk of developing AKI, shorten hospital stays, and guide individualized post-procedural care, including decisions about repeat ESWL or alternative therapies. Despite promising findings, the current body of evidence is limited by heterogeneity in biomarker selection, sample timing, stone size and ESWL parameters such as shock wave frequency and energy levels. Novel biomarkers can provide comprehensive monitoring post ESWL, however, variability in biomarker responses and limited long-term outcome data mean they should supplement rather than replace conventional renal function tests and clinical judgment.

## Appendices

Appendix A: search strategy for various databases

**Table 5:** Search strategy for the databases

Data Base	Search Strategy	No. of articles
PUBMED:	("Extracorporeal Shock Wave Lithotripsy"[Mesh] OR "shock wave lithotripsy" OR ESWL) AND ("Biomarkers, Urinary"[Mesh] OR "urinary biomarker*" OR "urine marker*" OR biomarker* OR NGAL OR "neutrophil gelatinase-associated lipocalin" OR KIM-1 OR "kidney injury molecule-1" OR IL-18 OR "interleukin-18" OR NAG OR "N-acetyl-β-D-glucosaminidase" OR "cystatin C" OR netrin-1 OR MCP-1 OR "monocyte chemoattractant protein-1" OR lncRNA OR mRNA OR microRNA) AND ("Acute Kidney Injury"[Mesh] OR "acute kidney injury" OR AKI OR "renal injury" OR "kidney injury" OR "renal tubular injury" OR "renal function" OR "renal damage")	47
COCHRANE	#1 "shock wave lithotripsy" OR ESWL #2 urinary biomarker* OR NGAL OR KIM-1 OR IL-18 OR NAG OR "cystatin C" OR netrin-1 OR MCP-1 OR lncRNA OR mRNA OR microRNA #3 "acute kidney injury" OR AKI OR "renal injury" OR "kidney injury" #4 #1 AND #2 AND #3	52
EMBASE	('extracorporeal shock wave lithotripsy'/exp OR 'shock wave lithotripsy' OR ESWL) AND ('urinary biomarker'/exp OR 'urinary biomarker*' OR 'urine marker*' OR biomarker* OR NGAL OR 'neutrophil gelatinase associated lipocalin' OR KIM-1 OR 'kidney injury molecule 1' OR IL-18 OR NAG OR 'n acetyl beta d glucosaminidase' OR 'cystatin c' OR netrin-1 OR MCP-1 OR lncRNA OR mRNA OR microRNA) AND ('acute kidney injury'/exp OR 'acute kidney injury' OR AKI OR 'renal injury' OR 'kidney injury' OR 'renal tubular injury')	113
SCOPUS	TITLE-ABS-KEY("shock wave lithotripsy" OR ESWL) AND TITLE-ABS-KEY("urinary biomarker*" OR "urine marker*" OR NGAL OR "neutrophil gelatinase-associated lipocalin" OR KIM-1 OR "kidney injury molecule-1" OR IL-18 OR "interleukin-18" OR NAG OR "cystatin C" OR netrin-1 OR MCP-1 OR lncRNA OR mRNA OR microRNA) AND TITLE-ABS-KEY("acute kidney injury" OR AKI OR "renal injury" OR "kidney injury" OR "renal tubular injury" OR "renal function")	54
